# High-speed mosaic imaging using scanner-synchronized stage position sampling

**DOI:** 10.1117/1.JBO.27.1.016502

**Published:** 2022-01-24

**Authors:** Chi Huang, Vincent Ching-Roa, Yihan Liu, Michael G. Giacomelli

**Affiliations:** a University of Rochester, Department of Biomedical Engineering, Rochester, New York, United States; b University of Rochester, Institute of Optics, Rochester, New York, United States

**Keywords:** nonlinear microscopy, two-photon microscopy, mosaic imaging, laser scanning microscopy

## Abstract

**Significance:**

Two-photon and confocal microscopy can obtain high frame rates; however, mosaic imaging of large tissue specimens remains time-consuming and inefficient, with higher imaging rates leading to a larger fraction of time wasted translating between imaging locations. Strip scanning obtains faster mosaic imaging rates by translating a specimen at constant velocity through a line scanner at the expense of more complex stitching and geometric distortion due to the difficulty of translating at completely constant velocity.

**Aim:**

We aim to develop an approach to mosaic imaging that can obtain higher accuracy and faster imaging rates while reducing computational complexity.

**Approach:**

We introduce an approach based on scanner-synchronous position sampling that enables subwavelength accurate imaging of specimens moving at a nonuniform velocity, eliminating distortion.

**Results:**

We demonstrate that this approach increases mosaic imaging rates while reducing computational complexity, retaining high SNR, and retaining geometric accuracy.

**Conclusions:**

Scanner synchronous strip scanning enables accurate, high-speed mosaic imaging of large specimens by reducing acquisition and processing time.

## 1 Introduction

Applications such as pathology^1^^–^^3^ and imaging of cleared tissue specimens^4^^,^^5^ frequently demand high imaging rates to enable large volumes of tissue to be imaged in a reasonable time. For example, in applications such as intraoperative margin assessment in breast^6^^,^^7^ or skin cancer,^8^ the surgical margin may span thousands of square millimeters but may be available for imaging for only a few minutes before a diagnosis is required. Conversely, in applications such as tissue clearing,^9^ specimens can typically be imaged indefinitely, but the enormous volume of many cleared tissues still needs high imaging rates to be practical.

Laser scanning techniques such as confocal and two-photon microscopy obtain high resolution while providing the rejection of out-of-focus light, leading to sharp, well-defined axial sections.^10^ Furthermore, in the case of two-photon microscopy, the extremely low sensitivity to scattering enables imaging 100s to 1000s of microns into tissue^11^ while nondescanned detection maximizes photon collection efficiency.^12^ Unfortunately, photomultiplier tubes (PMTs) used for conventional fluorescence imaging typically limit imaging rates to at most a few megapixels per second, with higher imaging rates requiring a reduction in photons per pixel to avoid saturation. For example, a typical GaAsP PMT saturating at about 2 billion photons per second would have a maximum possible shot noise limited SNR of just 20 when operated at 5  MP/s. In reality, this SNR will be significantly lower due to excess noise resulting from photoelectron multiplication.^13^ Alternative detectors, such as photodiodes and avalanche photodiodes, can have higher saturation powers but have much lower gain, limiting sensitivity, and making them challenging to use with dim or photosensitive samples.

Recently, silicon photomultiplier (SiPM) technology has attracted attention as a low-cost and high-performance alternative to conventional detectors in fluorescence microscopy.^14^^,^^15^ Compared to PMTs, SiPMs are inexpensive, operate in direct light without damage, have higher quantum efficiency at red and NIR wavelengths, and have negligible excess noise. We evaluated SiPM detectors and determined that at higher imaging rates, they had superior sensitivity to conventional GaAsP PMTs.^16^ Subsequently, we designed open-source high-dynamic range detection electronics optimized for high throughput imaging, enabling more than an order of magnitude increase in photon throughput compared to PMTs.^17^ Finally, we demonstrated that this improvement in throughput could be directly translated into higher imaging speeds.

Although the high photon throughput of SiPM detectors enables high imaging frame rates, this does not directly translate into proportionally faster mosaic imaging of large specimens because at high imaging rates an increasingly large fraction of the total imaging time is spent translating the specimen between positions. To address this, previous work in light sheet,^18^ confocal,^19^^,^^20^ line-scan confocal,^21^ fluorescence microscopy,^22^ and HiLo microscopy^23^ has utilized so-called “strip mode” or “push broom” scanning wherein the specimen is translated at a constant velocity through a fixed line imaging position. By setting the translation speed proportionally to the line imaging time, lines of adjacent pixels along the translation axis can be assembled into seamless frames. The disadvantage of this approach is that the specimen must be accelerated to exactly the correct speed to avoid distortion along the translation axis. Typically, additional iterative postprocessing must be applied to compensate for errors in timing or velocity^19^ while some amount of imaging time is wasted while the specimen accelerates to the target velocity. Previously we developed a related technique, video frame mosaicking, where precision position encoders were used to measure the 3D position of video frames in a live microscope imaging session and then reconstruct a mosaic from video frames.^7^ In contrast to the previous work, this approach is noniterative; the location of all pixels is known *a priori* with subwavelength accuracy and reconstruction directly repositions each pixel using the position encoder feedback enabling higher resolution, improved accuracy, and faster computation. Although focused on 2D video frames, this approach can be generalized to the stitching of strip images.

In this paper, we combine two-photon microscopy, high-dynamic range SiPM detection electronics, and position encoders synchronized to scanning to accelerate large area tissue mosaic imaging. Scanner-synchronous readout of position encoders is used to determine the location of each pixel in a mosaic with subwavelength accuracy, enabling simple and highly efficient dewarping of images acquired from rapidly moving and accelerating tissue specimens. We demonstrate that this approach can allow higher efficiency mosaic imaging by enabling imaging of specimens moving at a nonconstant velocity while improving accuracy and resolution.

## 2 Materials and Methods

### 2.1 System Design

#### 2.1.1 Synchronous sampling of system and scanner position

In conventional mosaic imaging using a laser scanning microscope and resonant scanning, a line trigger is generated by the scanner to mark the start of each line of pixels. This trigger is then used to synchronize the sampling of pixels by the to the motion of the scanner. The resonant trajectory of the scanner can be dewarped by resampling pixels to be linear in position using the arccosine function and the line trigger. The slow axis mirror then advances one pixel width and begins capturing the next line of pixels until the frame is complete. Unfortunately, this conventional approach is inefficient for mosaic imaging, with long periods of dead time between frames while the specimen is translated to a new position. Furthermore, frames are subject to optical aberrations, vignetting, distortion, and finite translational accuracy. Some amount of frame overlap is then required for computational coregistration and image stitching, which further slows down mosaic acquisition and postprocessing.

Mosaic imaging can be significantly accelerated by operating in “strip” scanning mode, where the specimen is translated at a constant velocity, advancing one pixel width per line. Because the specimen moves at constant velocity with pauses in imaging only at the end of each image “strip,” the imaging duty cycle can be increased. However, accelerating the specimen to constant velocity requires time, during which pixels are distorted. Furthermore, truly constant velocity is extremely difficult to achieve and so typically, some distortion is present due to nonuniform translation of the specimen, resulting in geometric distortion and loss of spatial resolution. Previous work has addressed this using computational methods to stitch the distorted stripes by relying on features from adjacent strips to stitch in spite of geometric distortion,^24^ which reduces geometric distortion but typically has some loss of resolution or residual distortion. This limitation can be overcome by recognizing that the fast axis line trigger approach can be generalized to two- or three-dimensional scanning if the line trigger is used to read out the stage position at the start of each line. With a list of line coordinates, the same resampling algorithm used to remove resonant distortion can remove slow axis distortion by resampling each fast axis line to be uniformly spaced. Thus the need to accelerate to constant velocity before imaging is eliminated.

**Fig. 1:**
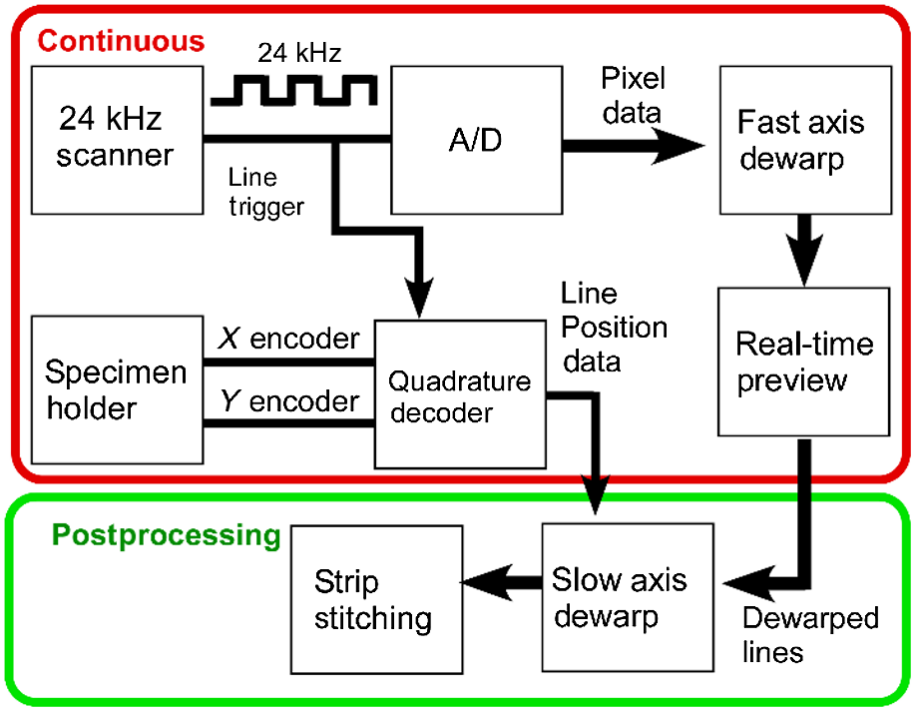
Synchronous sampling of a resonant scanning two-photon microscope and translation stage. The X/Y coordinates of each resonant line are recorded. After interpolation for fast axis resonant dewarping, a second interpolation is performed on along the slow (translation) axis, using the position sensor data to uniformly position each line.

We implemented synchronous stage sampling using a commercial 24 kHz (bidirectional) resonant scan head (LSK-GR12 Thorlabs, Inc.) and a two-axis brushless DC motor stage (MLS-203, Thorlabs, Inc.). The stage exposes the analog output from its optical position encoders with a resolution of 100 nm. These were converted from balanced to single-ended signals using commercial converters (C46, CNC4PC) and then sampled using a PCIe-6323 (National Instruments), which also provides control of the slow axis scanner when not used in strip mode. Synchronous sampling was performed by routing the scanner line trigger to the sampling clock input on the PCIe-6323’s quadrature decoder (Fig. 1). Finally, the line trigger output was routed to a four-channel A/D. Optimized interpolation written using a 4-tap cubic Hermite polynomial function was used to perform resonant scanner dewarping at over 400  MP/s on a single core of an Intel Core i5-9600k processor. Although both interpolation steps could be performed in real time on a single processor core, the slow axis interpolation was performed in postprocessing for simplicity and because the high line rate (24 kHz) means that lines are only on the screen for milliseconds before being overwritten making distortion-free real-time visualization relatively less important. To ensure that the 100 nm resolution of the stage encoders did not introduce numerical errors, piecewise linear fitting through the highly oversampled position data was used to calculate the starting position of each line with subnanometer resolution.

#### 2.1.2 Detector design

Four-channel detection was implemented using SiPM technology with current-domain pole zero cancellation described previously^17^ using the OpenSiPM design (https://github.com/OpenSiPM/). To optimize detection, the two shorter wavelength channels used blue-enhanced SiPMs (S14160-3015, Hamamatsu Photonics) with peak sensitivity at 460 nm, while the longer channels used red-enhanced SiPMs (S14420-3025) with peak sensitivity at 600 nm. Due to the smaller microcell size (15  μm), the blue channels have a theoretical saturation power greater than 400 billion photons per second, but this was limited to ∼90 billion photons per second by the bias generator. The red channels with 25  μm microcells saturate at 40 billion photons per second.

#### 2.1.3 Microscope design

All imaging was performed using a custom built two-photon microscope with a 16×/0.8  NA water immersion objective (CFI LWD Plan Fluorite, Nikon). Excitation was provided by an ytterbium fiber laser (YLMO-2W, Menlo Systems) with <150  fs, 1040 nm pulses at 100 MHz. In frame mode, the slow axis of the Thorlabs scan head was used, while in strip mode it was locked at the center position. In both modes, individual resonant scanner lines were sampled at 79.872 MHz (3328 times the resonant scanner bidirectional frequency) and then interpolated to generate 2048 linearly spaced pixels at a pitch of 260 nm.

### 2.2 Specimen Collection

Discarded human tissue specimens not required for diagnosis were acquired under a protocol approved by the Research Subjects Review Board. Discarded, fixed S100a4-Cre; Rosa-Ai9 mouse tendon samples from mice sacrificed by other labs were obtained for imaging.

#### 2.2.1 Specimen preparation

Human tissue specimens are sectioned into thin 75-μm sections then mounted onto glass slides after frozen section procedures. Human tissues were stained with 40  μg/ml acridine orange (A1301, ThermoFisher) and 40  μg/ml sulforhodamine 101 (#80101 Biotium) in acidic 70% EtOH (sodium acetate buffered pH=4.8) for 2 min following a 30-s wash in phosphate buffered saline (PBS). Fixed S100a4-Cre; Rosa-Ai9 mouse tendon were stained with 10  μM TO-PRO-3 iodide (T3605 ThermoFisher) in 70% EtOH for 5 min followed by a 30-s wash in PBS.

### 2.3 Image Coregistration

Pixels on the commercial scan head were anisotropic due to slightly larger scan angle on the resonant scanner compared to the galvanometer scanner. This was compensated for by measuring the pixel pitch with beads on both axes and resizing each frame to be isotropic. In addition, the X−Y axes of the translation stage and the resonant galvanometer scanner were misaligned by ∼0.8  deg. As a result, strip scans sheared by 0.8 deg, and mosaic frames were rotated 0.8 deg relative to the translation axis. This was compensated for by shearing each strip and rotating each frame accordingly.

## 3 Results

### 3.1 Evaluation of Geometric Distorting

**Fig. 2:**
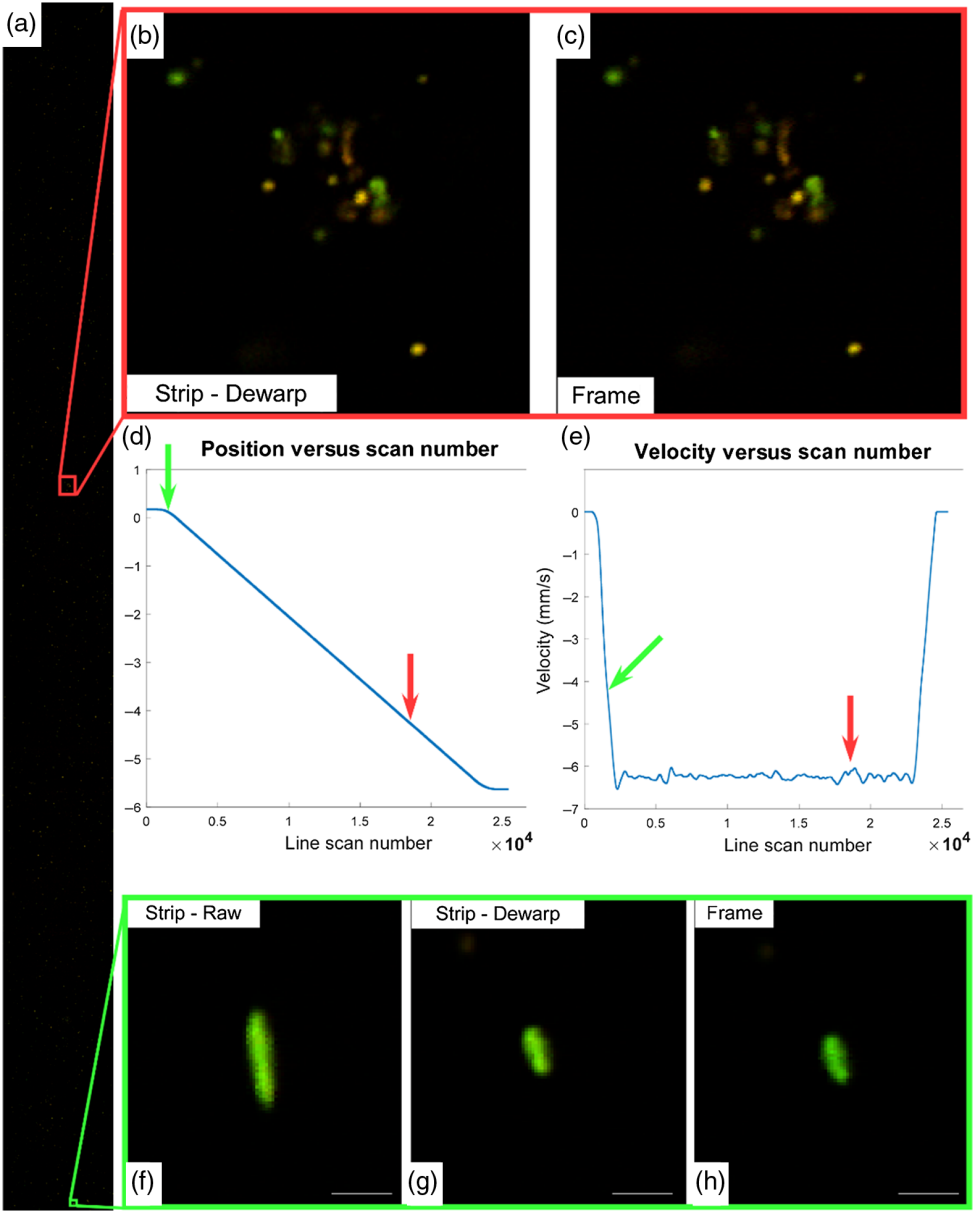
(a) 5.798×0.533  mm strip scan of a phantom with 1 to 10  μm multicolor beads acquired in 0.98 s. A cluster of beads imaged using (b) strip mode and (c) frame mode showing identical features and appearance. Images are recorded during the zero-acceleration phase of the scan. (d) Position and (e) velocity versus resonant scan number for the strip mode scan. Colored arrows represent the locations along with the scan of frames B and F. The acceleration phase lasts for 58 ms reaching an average velocity of 6.25  mm/s, equating to 260-nm pixel width. (f) Raw image data of a bead imaged during the acceleration phase of the strip mode scan. The same bead imaged with the distortion removed (g) and in frame mode (h) demonstrated an identical appearance. Scale bar: 5  μm.

To evaluate accuracy, mosaic images were acquired of multicolor fluorescent beads in PDMS. Specimens were first imaged in strip mode and then immediately reimaged in frame mode at identical excitation power. Figure 2(a) shows a representative strip. Figures 2(d) and 2(e) show the position and velocity, respectively, of each resonant scan in the strip with the location of subsequent subframes marked with arrows. Figures 2(b) and 2(c), acquired at the constant velocity phase of the strip, show that beads imaged in both modes are qualitatively identical. Conversely, Figs. 2(f)–2(h) show beads acquired during the acceleration phase, which lasted for 58 ms. The raw data [Fig. 2(f)] show the distortion of the bead size characteristic of acceleration. Figure 2(g) shows the same data with interpolation using the position encoder data, confirming that distortion is removed and that the bead is qualitatively identical to frame mode (F).

**Fig. 3:**
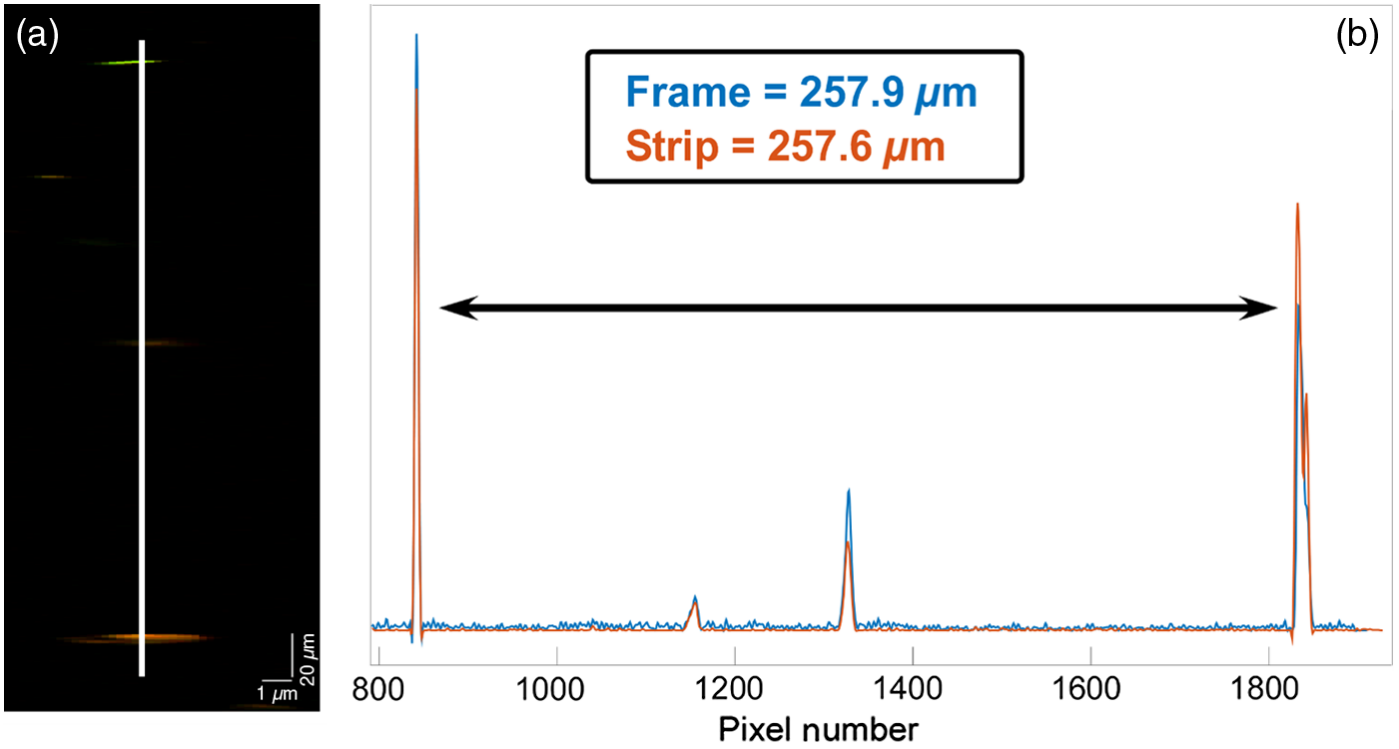
(a) Beads from the strip in Fig. 2. (b) The cross section indicated in (a) with the distance between two beads measured in frame mode and strip mode. Distances agree to less than the optical resolution (error of 1 pixel, 260 nm, or 0.1%), within error being contributed by barrel distortion in frame mode, uncertainty about the exact scanner field size, drift between images, and the optical encoder accuracy.

To quantitatively access accuracy, a pair of individual beads aligned along with the same lateral pixel and present within a single frame was selected from the same phantom, and the distance between them was measured in both frame and strip modes. Figure 3 shows a measure of 257.6  μm in strip mode and a distance of 257.9  μm in frame mode, a difference of 1 pixel or 260 nm. The discrepancy between these measurements results from the combined uncertainties of the stage position encoder (and so the accuracy of strip mode imaging), uncertainty about the exact aspect ratio of each pixel when operated in frame mode, geometric distortion in the scanner optics when operated in frame mode, and vibrational and focal drifts during ∼60  s that elapsed between measurements. Although lens distortion and focal drift are probably the largest error terms, the total combined uncertainty of both methods is less the optical resolution and therefore negligible even at large distances.

### 3.2 Imaging Tissue Specimens

**Fig. 4:**
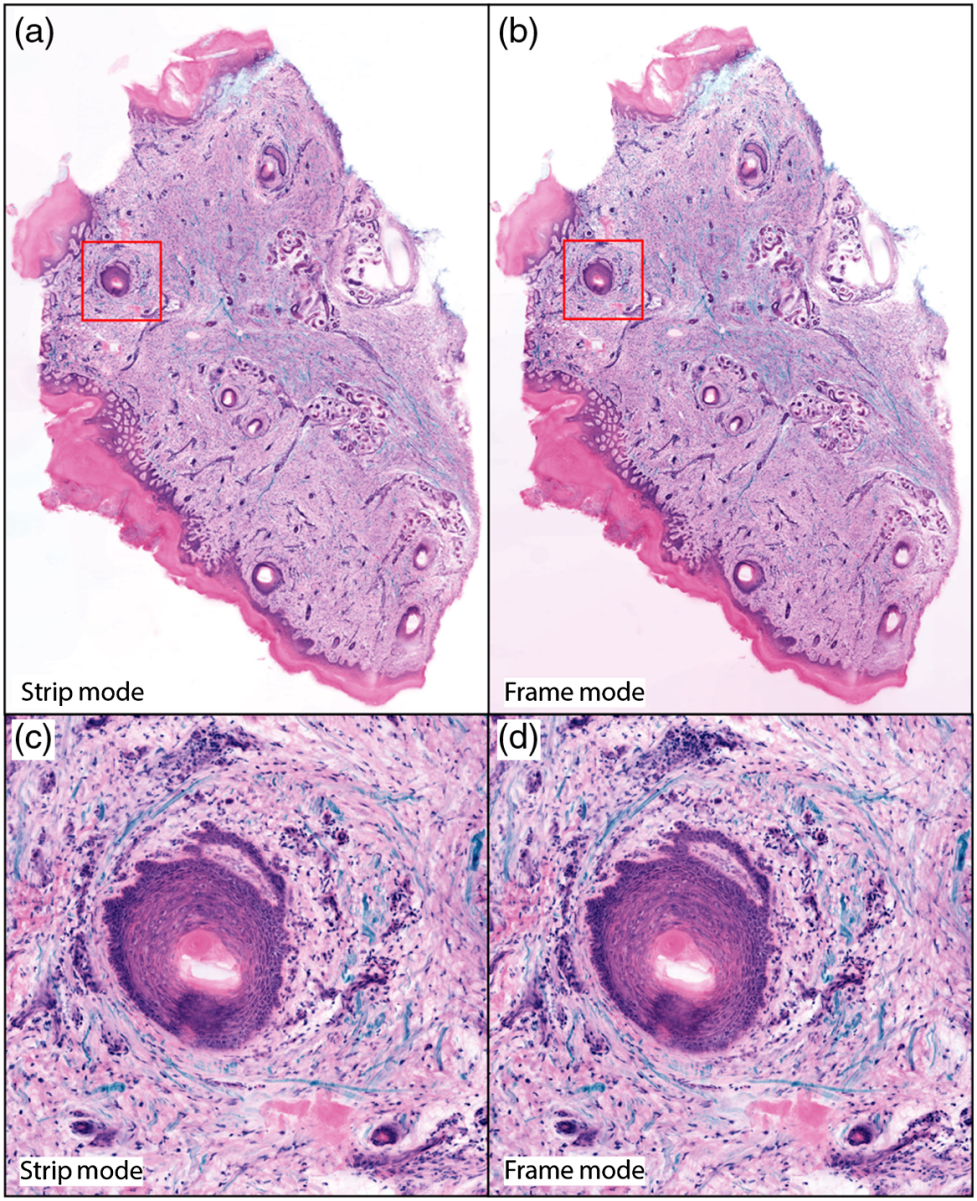
Human skin excision rendered as virtual Masson’s trichrome stains using the algorithm in Ref. 25 with acridine orange rendered as hematoxylin, sulforhodamine 101 rendered as eosin, and SHG rendered as methyl blue. Image acquisition using (a) strip mode and (b) frame mode. (c), (d) Enlarged regions show that microscopic features appear identical in strip and frame mode. Full resolution link: Ref. 26.

**Fig. 5:**
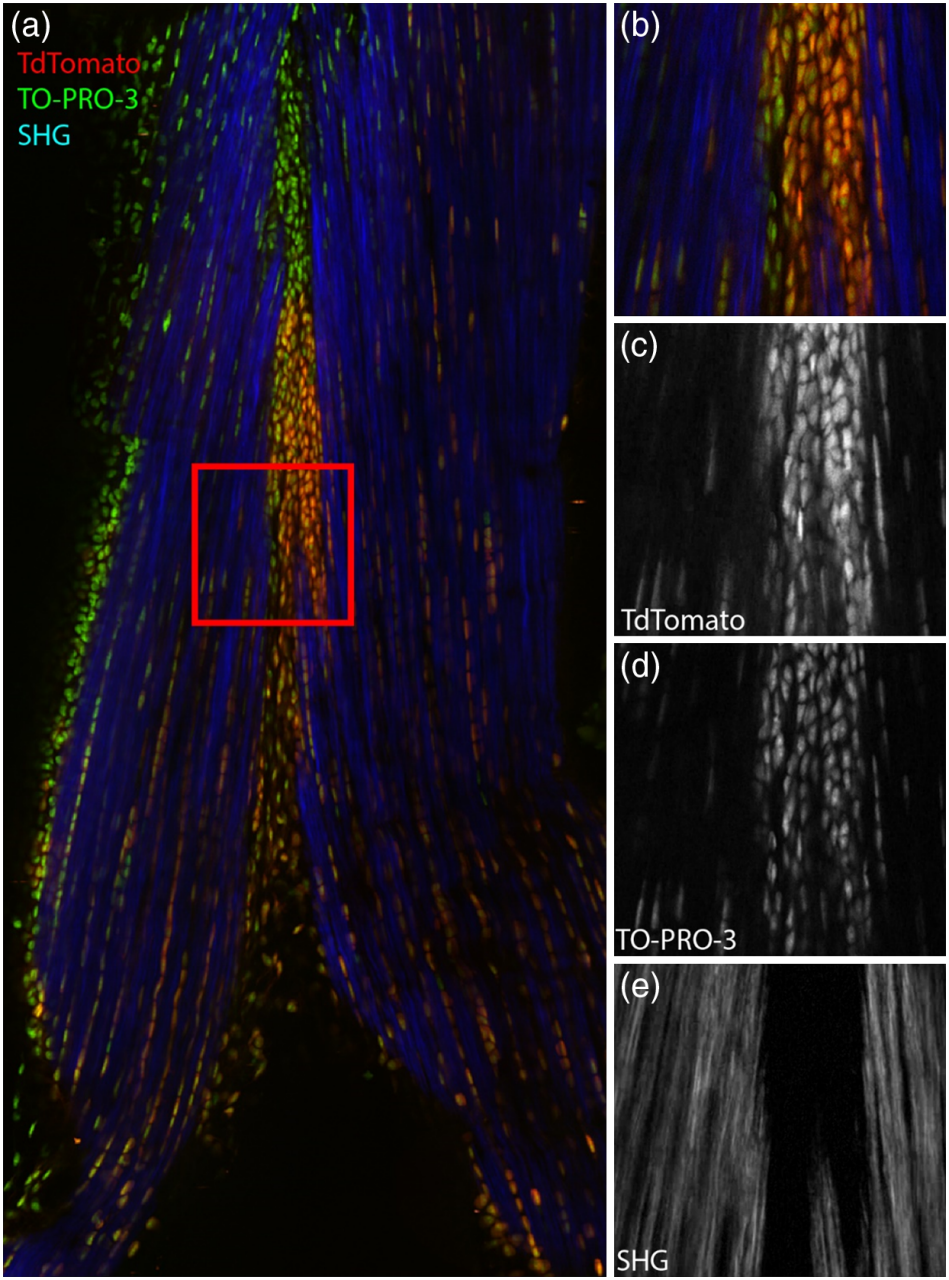
(a) S100a4-Cre; Rosa-Ai9 mouse tendon expressing TdTomato and labeled with TO-PRO-3 (DNA). Specimens were excited at 1040 nm. The enlarged region in (b) shows cells expressing TdTomato surrounded by collagen fiber. (c)–(e) The individual TdTomato, TO-PRO, and SHG channels. Full resolution link: Ref. 27.

Figure 4 shows imaging of a human skin specimen excised during Mohs surgery for suspected squamous cell carcinoma. Data were acquired using three channels, enabling virtual Masson’s trichrome staining where the second harmonic generation (SHG) is overlaid with the virtual H&E staining^25^ as blue fiber stain. The data are shown acquired in both frame and strip modes and appear visually identical in both modes. Figure 5 shows imaging of TdTomato expressing transgenic mouse tendon labeled using TO-PRO-3 for DNA imaged using the same configuration. Despite the peak imaging rate of nearly 50  MP/s, both the transgenic protein and SHG show good SNR.

### 3.3 Imaging Rate

**Fig. 6:**
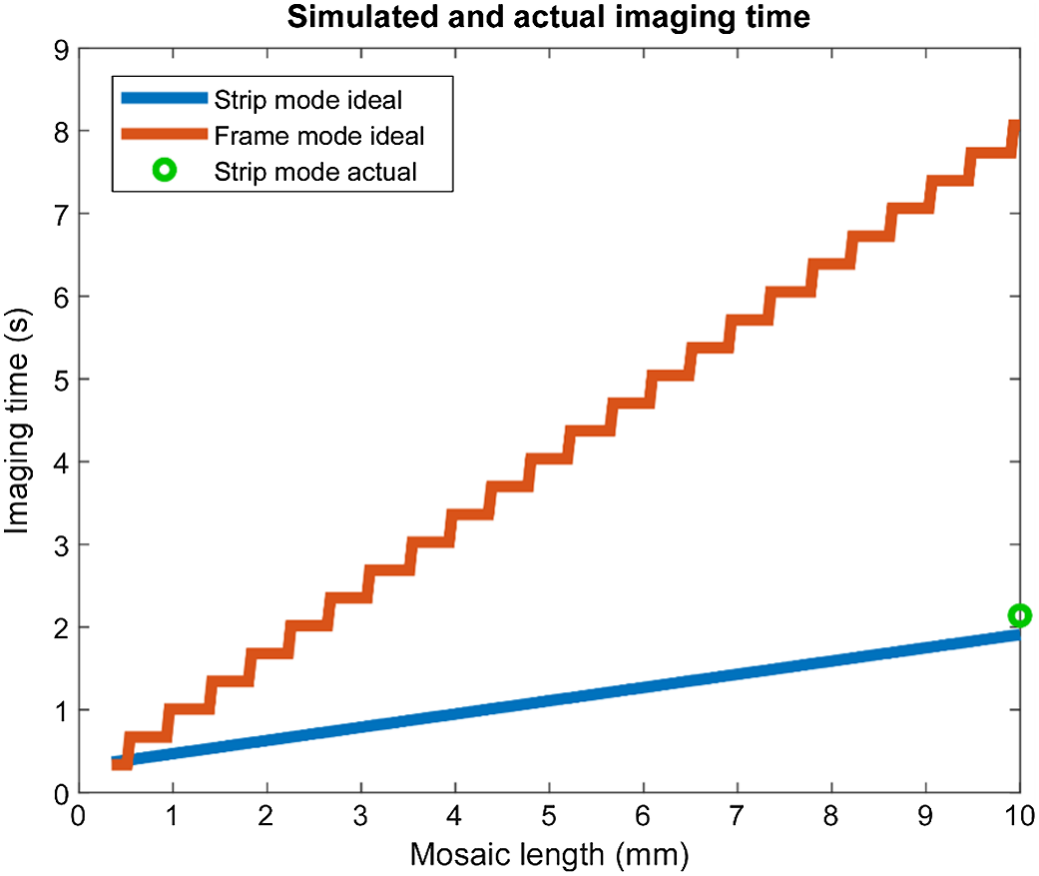
Simulated and actual imaging rates for a mosaic row at 260 nm pixel pitch. Both strip and frame mode assume a single 250 ms translation required to move to the starting location; thus the times required for an N row mosaic are N times the values given here. Strip mode has an absolute advantage except for the trivial case of a single-frame mosaic.

Mosaic imaging rates are determined by two parameters: the time required to acquire each image and the total time necessary to move the specimen, settle at a new location and rearm for the subsequent acquisition. In both modes, the microscope scanner operates at the same rate, producing 24,000 lines of 2048 pixels per second (49.1  MP/s). Similarly, moving the stage between frames or between strips takes approximately 250 ms. In principle, a 2048×2048 frame with 20 flyback cycles requires 0.086 s for acquisition and a further 250 ms for translation for a total of 336 ms per frame. For a 532-μm field of view and assuming 20% overlap to enable stitching, acquiring a row of frames 1 cm long would require 7.5 s. For a strip, the calculation is more complex because the acquisition time depends on the length of the strip and the rate of acceleration. At the start and end of each strip, acceleration lasts for 58 ms, during which the stage advances 175  μm for an average velocity of 3.0  mm/s, and thereafter, it advances 6.25  mm/s. Thus the imaging time is 116 ms for the first 350  μm and 160 ms per mm thereafter. Therefore, a 1-cm long strip has a theoretical acquisition time of 1.7 s with a further 250 ms for translation for a total of 1.95 s, or 4.51 times as fast as a frame acquisition. The relationship between row length and imaging time is depicted in Fig. 6.

In reality, it is not possible to reach ideal imaging rates because real systems require time to arm acquisitions, and copying data takes a finite time. Although we utilize multiple CPU cores to perform acquisition, stage control, and saving data to disk asynchronously, we could not fully hide all processing latency and measured a lower imaging rate, principally due to the time required to rearm the A/D between acquisitions. We measured 2.14 s per 1 cm in strip mode and 11.8 s in frame mode, 91% and 63% of the theoretical optimal times, respectively (Table 1). Although the same software was used in both cases, efficiency is higher in strip mode because the acquire, copy, move, and rearm cycle happens only once per strip and so incurs the constant software overhead only once per strip instead of once per frame. The overall imaging rate increase obtained by the real software implementation was 5.52 times faster.

**Table 1:** Experimental imaging rates at 260  nm×260  nm pixel pitch.

	Time for 1 cm row	Time per $1\;{\mathrm{cm}}^{2}$	Average pixel rate
Frame mode	11.81 s	259.8 s	6.67 MP/s
Strip mode	2.14 s	47.1 s	36.8 MP/s
Ratio		0.182×	5.52×

## 4 Discussion

Mosaic imaging is widely used in numerous areas of life sciences and medicine, but most literature addressing it aims to optimize imaging rates rather than minimize the time wasted between images. The most common approach, tile-based mosaicking, is fundamentally inefficient except at very low frame rates where the translation time can be neglected. For most microscopy applications, however, imaging times are comparable or smaller than the translation time, making tile-based mosaics a poor choice. Simultaneously, the need to acquire overlapping pixels on both axes results in more computationally expensive stitching after acquisition. Previous work using strip-based scanning has focused on the precision translation of specimens, iterative computational approaches to remove artifacts resulting from nonuniform translation or stitching at reduced resolution.^18^^,^^21^^,^^28^ However, this approach has limitations, including longer processing time, less efficient use of imaging time, and reduced overall accuracy both at small and larger scales. Conversely, for slide scanners, the use of line scan cameras with shutters synchronized to stage motion is widely used and known to provide high resolution and low distortion.^29^ We build upon this approach to enable higher resolution and more efficient use of imaging time in laser scanning microscopy where synchronization of a camera shutter is not possible. We show that high (subwavelength) accuracy is straightforward to obtain, even at extremely high imaging rates. Simultaneously, our approach is computationally simple enough that it is feasible to dewarp strips in real time, and while individual strips still need to be stitched into mosaics, the low distortion and need to perform stitching on only one axis greatly accelerates processing as compared to the frame mosaic case.

We have shown that the absolute accuracy over large distances agrees with distances measured within a single frame to within 260 nm. In reality, the true error is likely much smaller because distance measured within images obtained from laser scanning intrinsically has geometric distortion due to the microscope objectives and scan lenses. Operation in strip mode, which does not scan along the slow axis, has no such distortion along the translation axis. Similarly, measurement of small beads shows excellent (subpixel) agreement of their width between individual frames and mosaic strips. Thus accuracy is high relative to the optical resolution of the 0.8-NA microscope objective, suggesting that this approach should generalize to even higher resolution imaging. As expected from the bead data, imaging of both transgenic animal and human tissue specimens was possible at high speed and with virtually identical image results to conventional mosaicking, except that the imaging time was dramatically reduced.

The theoretical efficiency limit for our stage and frame rate, when operated in frame mosaic mode with 20% frame overlap, is 21% (that is, about one-fifth of the time is spent imaging unique pixels) due to the time that must be spent translating the stage and time spent imaging redundant pixels to enable stitching. While in principle faster stages or more advanced stitching code could improve this figure, this would be subject to rapidly diminishing returns due to mechanical limits and the contribution of small software and synchronization delays that become proportionally larger the faster translation becomes due to Amdahl’s law. Conversely, for a 1-cm-long strip sampled at 260 nm pixel pitch, the theoretical limit is 82% and asymptotically approaches 100% as the strip length increases. Furthermore, this advantage becomes larger as higher resolution objectives are used because in contrast to frame mosaicking, the strip velocity decreases with pixel pitch, resulting in a smaller relative contribution from the stage translation between strips. In our specific implementation, which was based on lightly modified software for frame mosaicking, we obtained 91% of the theoretical maximum imaging rate for a 1-cm strip (75% of total time spent imaging unique pixels).

In addition to the improvement in imaging rate and reduction in optical distortion, another advantage of the method proposed here is that the overall complexity, in both software and hardware, is low. We were able to retrofit a commercial Thorlabs stage and scanner by connecting the stage’s existing position encoders to the existing DAQ card included with the scanner. Software changes involved configuring a rectangular acquisition with the slow axis galvanometer mirror disabled and the stage set to trigger motion off of the frame trigger. Beyond the addition of a cable and an inexpensive balanced to a single-ended converter, no additional hardware was required. Similarly, the software required is extremely simple, and the existing frame mosaic acquisition software was significantly more efficient when operated in strip mode. After the acquisition, the code used for resonant dewarping on any resonant scanning microscope can be used to dewarp strips. Although we did not implement this in real time, it would be straightforward due to the low computational complexity. Similarly, stitching mosaics from strips was much faster than from frames, with Fig. 4 requiring more than 6 times longer to stitch the frame mosaic as compared to the strip mosaic due to the greatly reduced number of overlapping pixels and reduced distortion.

## 5 Conclusion

Scanner-synchronous strip scanning enables high-speed mosaic imaging of large specimens by continuously translating specimens during imaging. By eliminating the requirement for constant velocity translation, overall imaging rate and accuracy are improved while computational complexity is decreased. We have shown that images obtained by both methods were quantitatively and qualitatively similar, but acquisition and processing times were reduced by more than fivefold as compared to conventional frame-based mosaic stitching.

## Code, Data, and materials Availability

Links are provided for full-resolution images.
